# Revealing the architecture of the photosynthetic apparatus in the diatom *Thalassiosira pseudonana*

**DOI:** 10.1093/plphys/kiab208

**Published:** 2021-05-04

**Authors:** Rameez Arshad, Claudio Calvaruso, Egbert J Boekema, Claudia Büchel, Roman Kouřil

**Affiliations:** 1 Department of Biophysics, Faculty of Science, Centre of the Region Haná for Biotechnological and Agricultural Research, Palacký University, Olomouc 78371, Czech Republic; 2 Electron Microscopy Group, Groningen Biomolecular Sciences and Biotechnology Institute, University of Groningen, Groningen 9747AG, The Netherlands; 3 Institute for Molecular Biosciences, Goethe University of Frankfurt, Frankfurt 60438, Germany

## Abstract

Diatoms are a large group of marine algae that are responsible for about one-quarter of global carbon fixation. Light-harvesting complexes of diatoms are formed by the fucoxanthin chlorophyll a/c proteins and their overall organization around core complexes of photosystems (PSs) I and II is unique in the plant kingdom. Using cryo-electron tomography, we have elucidated the structural organization of PSII and PSI supercomplexes and their spatial segregation in the thylakoid membrane of the model diatom species *Thalassiosira pseudonana*. 3D sub-volume averaging revealed that the PSII supercomplex of *T. pseudonana* incorporates a trimeric form of light-harvesting antenna, which differs from the tetrameric antenna observed previously in another diatom, *Chaetoceros gracilis*. Surprisingly, the organization of the PSI supercomplex is conserved in both diatom species. These results strongly suggest that different diatom classes have various architectures of PSII as an adaptation strategy, whilst a convergent evolution occurred concerning PSI and the overall plastid structure.

## Teaser

The antenna organization of photosystem II in the diatom *Thalassiosira pseudonana* strongly differs from *Chaetoceros gracilis*, while the architecture of the photosystem I antenna remains the same.

## Introduction

Diatoms, a large group of marine algae, have enormous importance for global ecological systems on our planet. It has been estimated that they represent about 40% of the marine biomass and their contribution to the overall Earth’s primary production is about 25% (Falkowski et al., 1998). In the light of the current advances in biotechnology, diatoms are also considered as a potentially valuable source of dietary supplements, antibiotics, and pharmaceutically active substances (Bozarth et al., 2009). Despite their crucial role in the Earth’s carbon cycle, very little is known about their fundamental metabolic pathways and about the organization of their photosynthetic apparatus.

Diatoms originated in a secondary endosymbiosis event between a red algal ancestor and a eukaryotic cell (Bhattacharya et al., 2007), which predetermines the structure and properties of their plastids. Diatom plastid envelope has four membrane layers and their thylakoids are arranged in a three-band structure (Bedoshvili et al., 2009). Even though the thylakoids lack the grana–stroma organization typical for vascular plants, there is still a certain degree of spatial segregation of photosystems I (PSI) and II (PSII): PSII is localized predominantly in appressed membranes, whereas PSI is concentrated in thylakoid membranes exposed to the stroma (Pyszniak and Gibbs, 1992; Flori et al., 2017; Levitan et al., 2019).

The light-harvesting antenna system of diatoms differs from green plants and consists of fucoxanthin–chlorophyll a/c protein (FCP) complexes (Büchel, 2020). Diatoms have a large set of genes encoding different light-harvesting complex (Lhc) proteins, which can be classified into three main families: Lhcf, Lhcr, and Lhcx. Although the functional association of individual families with PSII and PSI complexes is not well understood, it has been assumed that Lhcf is the major group of light-harvesting antenna proteins for both PSs, Lhcr proteins are preferentially connected to PSI (due to their similarity with red algal antennae serving PSI) and Lhcx proteins have a function in stress response and photoprotection (Büchel, 2020). These protein families also differ in their ability to form various oligomeric structures. So far FCP monomers, dimers, trimers, and tetramers have been observed (Nagao et al., 2019; Pi et al., 2019; Wang et al., 2019; Büchel, 2020).

Although there has been an enormous advance in our understanding of the architecture of the photosynthetic apparatus in green algae and land plants (Kouřil et al., 2018; Cao et al., 2020), the information about the structure of photosynthetic complexes of diatoms is still very limited. The main reason appears to be the complexity of the chloroplast structure, which also makes the isolation of PSs in their native state very problematic (Schober et al., 2019). Cryo-electron tomography (ET) of thylakoid membranes in diatoms was performed very recently on *Phaeodactylum tricornutum* (Levitan et al., 2019). This study provided initial structural indications about spatial segregation of photochemically active and inactive PSII complexes in the membrane, which are separated from PSI complexes and clusters of ATP synthase (Levitan et al., 2019).

Recently, thanks to optimized isolation protocols, it was possible for the first time to use cryo-electron microscopy (EM) to obtain structural information about isolated PSII and PSI supercomplexes from one of the centric diatoms, *Chaetoceros gracilis* (Nagao et al., 2019; Pi et al., 2019; Nagao et al., 2020a, 2020b; Xu et al., 2020). Although the diatom PS core complexes appear to be similar to core complexes of other known photosynthetic species, their light-harvesting antennae seem to be unique. Instead of the trimeric light-harvesting (LHCII) antennae that are typical for green algae and land plants, PSII antenna complexes of *C. gracilis* consist of FCP tetramers (Nagao et al., 2019; Pi et al., 2019). The binding of these tetramers to the PSII core is supported by three monomeric FCP subunits (called FCP-D, -E, and -F). This is analogous to the situation in green algae and land plants, however, here the localization of the three monomeric subunits (Lhcb4, 5, and 6) is different. The organization of the light-harvesting antenna of PSI of *C. gracilis* shows also very atypical features. It consists of a highly variable number of FCP subunits, ranging from 16 to 24 copies, which can completely surround the core complex in several layers to form a giant supercomplex (Nagao et al., 2020a, 2020b; Xu et al., 2020).

A centric diatom *Thalassiosira pseudonana*, the first eukaryotic marine phytoplankton species whose genome has been fully sequenced (Armbrust et al., 2004), is generally used as a model organism. In our previous report (Calvaruso et al., 2020), we have isolated near-native supercomplexes of *T. pseudonana* and provided their in-depth proteomic characterization. Here, we extended this study by ET and sub-volume averaging to obtain information about the structure of PSII and PSI supercomplexes of *T. pseudonana* and about their spatial distribution in the thylakoid membrane. Although there appear to be many similarities between the photosynthetic apparatus of *T. pseudonana* and *C. gracilis*, surprisingly, we have also revealed several aspects in which they differ. While the organization of PSI supercomplexes in both diatom species shows common structural features, their PSII supercomplexes have a different architecture of the Lhcs. We have found that the antenna of *T. pseudonana* is formed by FCP trimers instead of tetramers, which were earlier observed in *C. gracilis*. This indicates that the evolutionary distance between these two species, which belong to two different subclasses of diatoms, is also reflected in the structure of their photosynthetic apparatus, especially in the arrangement and complement of antenna complexes of PSII.

## Results

### The 3D architecture of a thylakoid membrane vesicle

**Figure 1:**
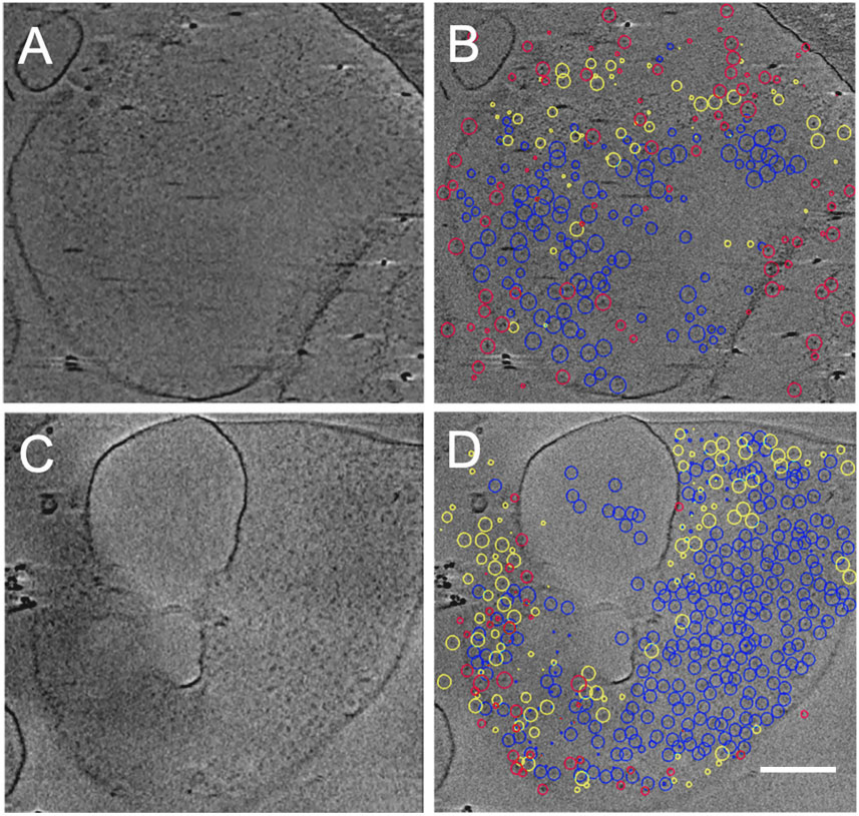
Cryo-ET of an isolated thylakoid membrane vesicle from *T. pseudonana*. A–D, Tomographic slices of a representative cryo-electron tomogram. A and C, tomographic slices represent the top and bottom layers of the thylakoid membrane vesicle. B and D, picking of the sub-volumes from the tomographic slices, which contain densities of PSII (blue circles), PSI (yellow circles), and ATP synthase (red circles) particles. The scale bar is 200 nm.

Cryo-ET was performed to visualize both the overall organization of thylakoid membranes from *T. pseudonana* and the main components of the photosynthetic apparatus embedded in the thylakoid membrane. As a thin specimen is required to obtain high resolution in a reconstructed tomogram (Crowther et al., 1970), we gently separated thylakoid membranes into individual vesicles, starting from near-intact plastids. Connections between stacked membranes were gently cleaved in a low ionic strength buffer with a low concentration of detergent. Tomographic tilt series of individual thylakoid vesicles were collected and subsequently aligned to generate a 3D tomogram. The reconstructed tomogram shows a thylakoid vesicle of a round shape with a diameter of about 1 μm (Supplemental Movie S1). Inspection of tomogram slices revealed the presence of densities of different protein complexes embedded in the thylakoid membrane (Figure 1). PSII supercomplexes were recognized by their typical densities resulting from their dimeric core complex, round-shape densities were attributed to the PSI core complex and ATP synthase was recognized due to its strong round density of the hydrophilic head sticking out of the thylakoid membrane (Supplemental Figure S1). Individual projections were analyzed by sub-volume averaging to confirm the identity of these densities (see below).

### Structural characterization of the PSII supercomplex

Sub-volume averaging of PSII particles from *T. pseudonana* revealed the presence of a PSII supercomplex of an uncommon shape*.* The isosurface model of PSII allowed us to distinguish features of the core complex, including the extrinsic subunits of the oxygen-evolving complex, and associated light-harvesting antenna (Supplemental Movie S2).

**Figure 2:**
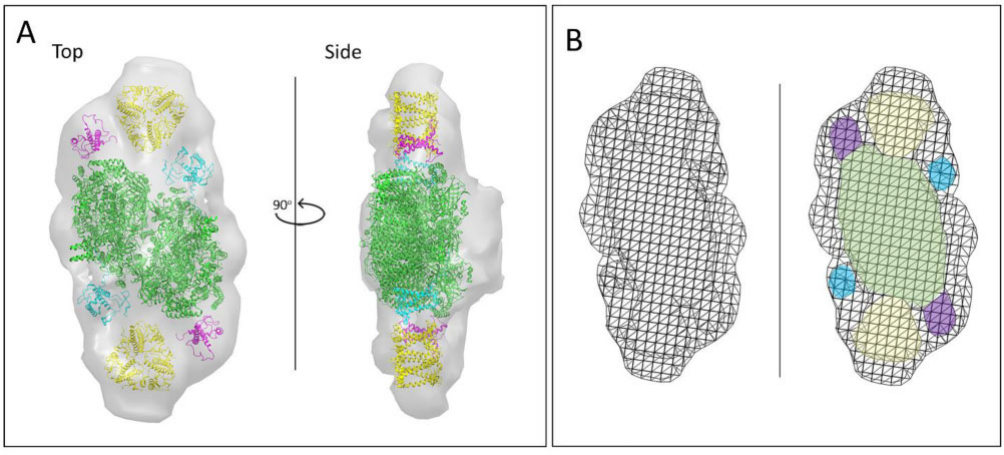
Structural model of the PSII supercomplex from *T. pseudonana* revealed by cryo-ET and sub-volume averaging. A, the top-view of the isosurface of the 3D map of the PSII supercomplex represents the view from the luminal side. The oxygen-evolving complex at the membrane–lumen interface is visible in the side-view. Densities representing the PSII core complex (in green) and monomeric antenna (FCPII-1 in cyan, FCPII-2 in purple) are fitted with *C. gracilis* PSII structure (PDB 6jlu), the trimeric form of FCP light-harvesting antenna is fitted with pea LHCII structure (in yellow; PDB 2bhw). B, mesh representation of 3D map of PSII supercomplex. The PSII core is depicted in green, monomeric antenna FCPII-1 and FCPII-2 proteins are visualized in cyan and purple, respectively, and the FCP trimer in yellow.

The structural model of the 3D map of the PSII supercomplex was created by fitting the PSII core complex from *C. gracilis* (Pi et al., 2019). Typical features of the PSII core complex were identified in the 3D volume of PSII, and thus the position of the core complex was unambiguously assigned (Figure 2). For the fitting of the light-harvesting antenna complexes, we generated a mesh view of our 3D map, which helped us to reveal the locations of individual components of the antenna system (Figure 2B). In the mesh view of the 3D map, were have clearly identified antenna trimers, denoted as FCPII-S, whose densities were also recognizable in the isosurface volume of PSII (Figure 2B). The ability of FCPs to form trimers was also confirmed by single-particle analysis of the FCP fraction (Supplemental Figure S2). In line with the mesh view, the remaining densities of the light-harvesting moieties were fitted by two monomeric antenna proteins. As the positions of the monomers in *T. pseudonana* are different compared to *C. gracilis*, it was not possible to identify them based on the correspondence between the models. Although the EM densities of FCPII-1 and FCPII-2 were best fitted with *C. gracilis* structures of FCP-D and FCP-E, respectively, we were not able to unequivocally assign the monomers to particular proteins, and therefore we named them FCPII-1 and FCPII-2.

The outcome of the cryo-ET and sub-volume averaging indicates that the PSII supercomplex of *T. pseudonana* consists of a dimeric core (C_2_), two trimeric antennae (S_2_), and four monomeric antenna subunits, forming a unique C_2_S_2_ supercomplex.

### Structural characterization of the PSI supercomplex and the ATP synthase

**Figure 3:**
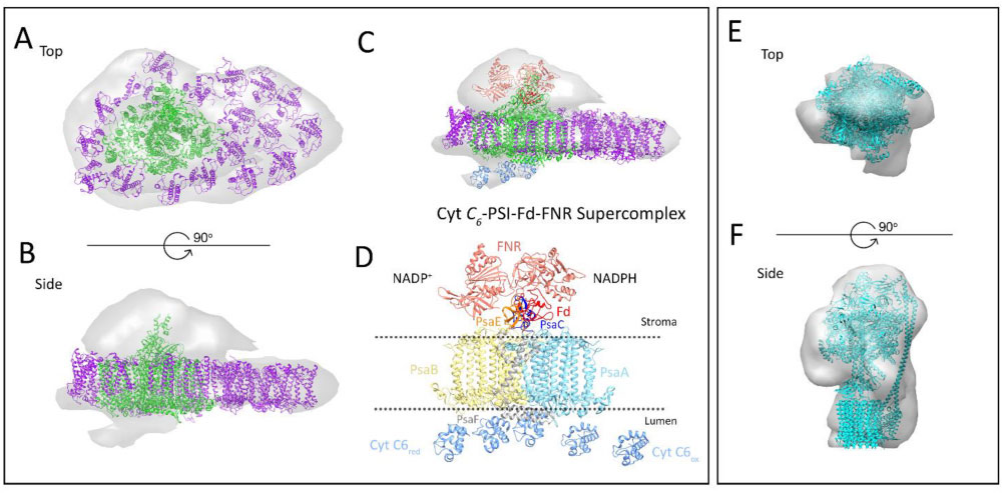
Structural models of the PSI supercomplex and the ATP synthase from *T. pseudonana* revealed by cryo-ET and sub-volume averaging. A–B, the top-view of the isosurface model of PSI supercomplex represents the view from the stromal side, whereas membrane extrinsic subunits of PSI are visible in the side view. Assignment of EM densities is based on the fitting of the high-resolution structure from *C. gracilis* (PDB 6ly5). The PSI core complex is indicated in green and FCPI subunits are in purple. C, side-view of the isosurface model shows the membrane-extrinsic densities. At the stromal side of PSI, the density is fitted with ferredoxin (in red) and two copies of the FNR complex (in salmon; PDB 1 gaq). At the luminal side, the densities are fitted with cyt *c_6_* (in light blue; PDB 3 dmi). D, A hypothetical model showing the association of FNR and cyt *c_6_* with the main subunits of the PSI supercomplex. PsaA, PsaB, PsaC, PsaE, and PsaF are shown in sky blue, yellow, blue, orange, and gray, respectively. Fd is shown in red, FNR in salmon, and cyt *c_6_* in blue. E–F, Top-view, and side-view of the isosurface model of ATP synthase fitted with the high-resolution structure of chloroplast ATP synthase (PDB 6 fkh).

Sub-volume averaging of selected PSI particles revealed a 3D map of a huge PSI supercomplex, which has typical features of the PSI complex previously observed in *C. gracilis* (Nagao et al., 2020a, 2020b; Xu et al., 2020). This complex is formed by the PSI core with 18 antenna subunits (Figure 3). Fitting of the obtained projection maps with the high-resolution cryo-EM structure of a giant PSI supercomplex from *C. gracilis* (Xu et al., 2020) demonstrated that they have a very high degree of similarity. The only distinct feature is the absence of six peripheral FCPI proteins at the PsaF and PsaL sides of the PSI core complex in *T. pseudonana* (Figure 3;Supplemental Movie S3). These FCP subunits are missing in our structure compared to the *C. gracilis* PSI structure published by Xu et al. (2020), whereas three more are present in comparison to the structure of PSI from the same organism shown by Nagao et al. (2020a, 2020b). These differences can be related either to different growth conditions or can be species-specific.

Interestingly, the isosurface of the 3D map of the PSI supercomplex revealed additional, membrane-extrinsic densities at both the stromal and luminal side of the PSI supercomplex. Since these extrinsic densities are visualized at the same isosurface threshold as the PSI volume, they should indeed represent real protein densities. As they are located close to the electron donor and acceptor sides of PSI, it is reasonable to expect that these densities represent some other components of the electron transport chain. Hence, these densities were fitted with ferredoxin-NADP^+^ oxidoreductase (FNR) at the acceptor side, and multiple copies of cytochrome (cyt) *c_6_* (a protein that replaces plastocyanin [Pc] in diatoms) at the donor side (Figure 3C). This hypothetical model shows the formation of a huge cyt *c_6_*-PSI-Fd-FNR supercomplex, which is able to mediate electron transfer.

As expected, sub-volume averaging of ATP-synthase particles resulted in the typical 3D map of ATP synthase (Figure 3, E and F). Both the catalytic head and the membrane intrinsic part of ATP synthase can be recognized, even under this rather limited resolution.

### Structural analysis of isolated PSII and PSI supercomplexes

**Figure 4:**
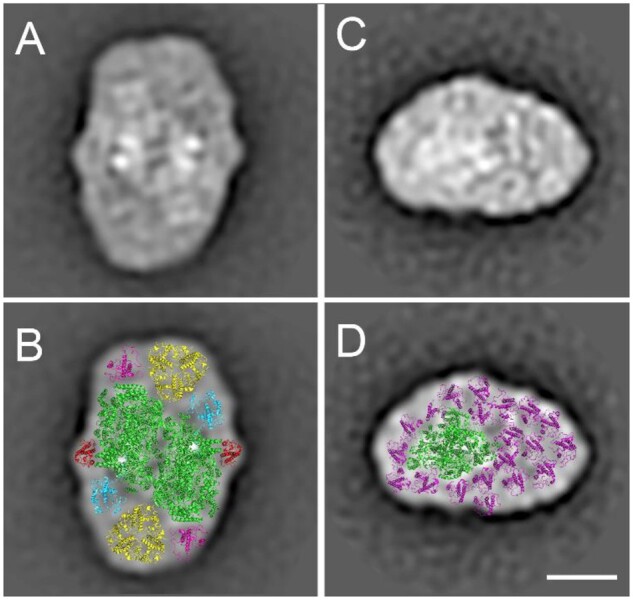
Structural models of the PSII and PSI supercomplexes from *T. pseudonana* revealed by EM and single-particle analysis. A and C, 2D projections of isolated and negatively stained PSII and PSI supercomplexes. B, Assignment of EM densities is based on the fitting with high-resolution structures. Densities representing PSII core complex and monomeric antenna are fitted with the PSII structure from *C. gracilis* (PDB 6 jlu). The trimeric form of the light-harvesting antenna is fitted with pea LHCII structure (PDB 2 bhw). A density close to the PSII core subunit PsbX (designated as FCPII-3) was fitted with *P. tricornutum* FCP monomer (PDB 6 a2w). PSII core complex is shown in green, FCPII-1 in cyan, FCPII-2 in purple, FCPII-3 in red, and trimeric FCP antenna (FCPII-S) is in yellow. D, the assignment of EM densities is based on the fitting with high-resolution structure from *C. gracilis* (Xu et al., 2020; PDB 6ly5). The PSI core complex is shown in green and FCP subunits are in purple. The scale bar is 10 nm.

Sub-volume averaging allowed us to construct 3D structures of PSII and PSI supercomplexes directly in the thylakoid membrane. To see how these in situ models are structurally related to isolated supercomplexes, we performed single-particle EM of the PSII and PSI particles isolated from *T. pseudonana* using sucrose gradient (SG) ultracentrifugation (Supplemental Figure S3). The image analysis revealed that PSII and PSI supercomplexes (Figure 4) are similar to structures obtained from sub-volume averaging of tomography data. A structural assignment of the 2D projection maps of PSII and PSI supercomplexes resulted in the construction of structural models, which are almost identical to the models obtained from cryo-ET and sub-volume averaging (Figures 2 and 3). However, in the 2D projections of PSII, a unique density (labeled as FCPII-3) was found at the periphery of the core complex (close to the core subunit PsbX). Surprisingly, this density was found to be too small to be fitted by either FCP-D, FCP-E, or FCP-F subunits from *C. gracilis.* Instead, we found that the density can be fitted by the most compact type of FCP monomer that was recently structurally characterized, PtLhcf4 of *P. tricornutum* (Wang et al., 2019). Albeit belonging to another species, this protein still shows a high sequence similarity of over 60% with many Lhcf proteins of *T. pseudonana*, suggesting a common structural arrangement.

Image classification of the 2D projections in the PSII and PSI datasets revealed the presence of smaller forms of both PSII and PSI supercomplexes (Supplemental Figures S4 and S5). As isolated large forms of PSII supercomplexes are known to be prone to disassembly, the appearance of the smaller forms of PSII suggests a gradual loss of light-harvesting antenna subunits, hence, reducing the particles to a PSII core complex with no antenna bound (Supplemental Figure S4, B–F). Image analysis of PSI particles indicated the presence of three different forms of PSI supercomplexes, which vary in the number of light-harvesting antenna subunits bound to the core complex (Supplemental Figure S5, A–C). Whether these forms represent physiologically relevant supercomplexes or products of a disassembly of the largest PSI supercomplex remain to be elucidated.

### Spatial distribution of the components of the thylakoid membrane

**Figure 5:**
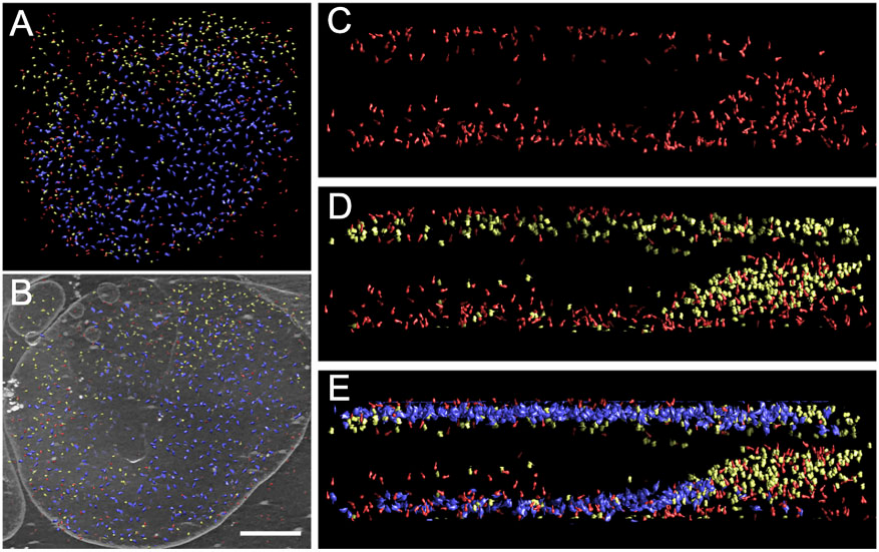
Spatial distribution of PSII, PSI, and ATP synthase particles in the vesicle of the thylakoid membrane from *T. pseudonana*. A, spatial distribution of PSII, PSI, and ATP synthase particles and B, their back-projection in the reconstructed thylakoid membrane. The scale bar is 200 nm. C–E, distribution of the particles viewed from the side of the membrane vesicle. Distribution of ATP synthase is shown in (C), PSI and ATP synthase in (D), and PSII, PSI, and ATP synthase in (E). PSII is shown in blue, PSI in yellow and ATP synthase is in red.

Positions of the individual particles selected for sub-volume averaging were tracked back in the reconstructed tomogram to visualize their spatial distribution in the thylakoid membrane (Figure 5;Supplemental Movie S4). The sub-volume analysis also enabled us to determine the orientation of analyzed particles in the membrane, which indicates our vesicle is a right-side-out vesicle, arguing against major re-arrangements of the membrane folding during preparation. The obtained model demonstrates that PSI, PSII, ATP synthase are at least partially spatially separated in different parts of the thylakoid membrane. Whereas the central region of the thylakoid vesicle is enriched in PSII particles, PSI and ATP synthase particles are preferentially localized on the edges of the vesicle. Separation is, however, not as strict as seen in grana and stroma thylakoids of land plants.

## Discussion

Over the last couple of years, the cryo-EM of isolated protein complexes has become a leading technique in the field of structural biology (Lyumkis, 2019). The cryo-ET further extends the potential of the cryo-EM technique. Although the resolution of cryo-ET is far lower than the resolution of single-particle cryo-EM technique, tomography can provide very valuable structural information about biological objects in situ, for example, at the level of the biological membrane, organelle, or a cell. Cryo-ET in combination with sub-volume averaging, which allows the computation of a 3D structure of a target of interest directly from the tomogram, has already been successfully used for the visualization of PSII core complexes embedded in isolated grana membranes of land plants (Daum et al., 2010; Kouřil et al., 2011). Here we have applied this approach to study the structures of PSII and PSI supercomplexes in situ in the diatom *T. pseudonana*. With the help of these advanced techniques, we were able to resolve in situ complete structures of the key components of the photosynthetic apparatus in this model centric diatom. For the first time, the structures of PSII and PSI supercomplex were solved together with their bound Lhcs directly from the tomogram, which gives us unique information about the organization of the supercomplexes in their natural environment—the thylakoid membrane.

### Structure of the PSII supercomplex in *T. pseudonana*

The overall organization of PSII supercomplexes in *T. pseudonana* is largely similar to PSII in land plants (Wei et al., 2016; Su et al., 2017; Van Bezouwen et al., 2017). The structural model of PSII supercomplexes from this diatom shows a dimeric core complex, where each core monomer binds two monomers (FCPII-1 and FCPII-2) and one trimeric antenna complex (FCPII-S; Figure 2). Surprisingly, the organization of the light-harvesting antenna in *T. pseudonana* clearly differs from what has been observed in another diatom species, *Chaetoceros gracilis* (Supplemental Figure S6), the most striking difference being the presence of FCP trimers instead of tetramers found in *C. gracilis* (Nagao et al., 2019; Pi et al., 2019). The occurrence of the trimeric antenna in PSII of *T. pseudonana* is well-supported by our previous biochemical analysis (Calvaruso et al., 2020) and by the following structural evidences. Contouring of the 3D electron density map of the PSII supercomplex obtained by sub-volume averaging revealed the binding of a trimeric antenna to the core complex (Figure 2), which was further supported by the single-particle analysis of isolated PSII supercomplexes (Figure 4, A and B). The trimeric form of FCP was further confirmed by the single-particle analysis of the isolated FCPs (Supplemental Figure S2).

The high degree of similarity between PSII models obtained by single-particle EM of isolated PSII supercomplexes and by cryo-ET and sub-volume averaging (Figures 2 and 4;Supplemental Figure S6) strongly indicates that the isolated PSII supercomplexes are very close to their native state. However, a close-up comparison of the two PSII models indicates a few minor differences. The structure of isolated PSII supercomplexes indicates a binding of one additional monomeric antenna protein, FCPII-3 (Figure 4), which was not clearly resolved in the 3D isosurface model of the PSII supercomplex (Figure 2). Further, FCPII-S trimers appear to be less tightly bound to the PSII core complex in the thylakoid membrane (Supplemental Figure S6). Our data also show that the binding position of the S trimer does not need to be occupied solely by the trimer, as in the case of green algae and land plants. Instead, in smaller PSII supercomplexes, the S trimer can be replaced by a monomeric antenna from the pool of FCPs (Calvaruso et al., 2020; Supplemental Figure S4, H–J). Although these complexes without trimeric antenna resemble PSII supercomplexes from another eukaryotic alga, the cryptophyte *Rhodomonas* (Kereïche et al., 2008), they could also be artifacts of the isolation and solubilization procedures.

Assuming that the FCPII-3 found in the isolated PSII is indeed a part of the supercomplex (Figure 4, A and B), our structural model can account for six different FCP polypeptides (FCPII-1, FCPII-2, FCPII-3, and supposedly three different polypeptides in FCPII-S). However, our recent biochemical analysis of PSII supercomplexes from *T. pseudonana* (Calvaruso et al., 2020) identified a larger number of proteins, with 8 Lhcf (TpLhcf1-7 and -11), 1 Lhcr (TpLhca2), and 1 Lhcx (TpLhcx6_1). A very similar antenna composition was also confirmed for the PSII SG fraction used here, that is, the presence of the pool of Lhcf proteins including TpLhcf1/2, and TpLhcx6_1 (Supplemental Figure S3). One plausible explanation of the discrepancy between the limited number of FCP proteins bound to PSII and the large variety of identified FCP proteins is the existence of different PSII sub-populations with heterogeneous antenna composition. This heterogeneity is probably related to variability in the subunit composition of the FCPII-S trimers. It has been reported that in centric diatoms grown under low light irradiation (similar to our growth conditions), the pool of free FCPs is formed mainly by Lhcf1-9 and Lhcx6_1 subunits (Grouneva et al., 2011; Gundermann et al., 2019), whereby TpLhcf8/9 is the major constituent of FCPs of higher oligomeric state and less abundant in FCP trimers. In PSII supercomplexes the subunits specific for FCP trimers were found previously (Calvaruso et al., 2020; and the absence of TpLhcf8/9 was confirmed here again). The FCPII-S might thus resemble the free pool of trimeric FCPs in subunit composition, implying a diversification of the trimeric antenna pool of PSII as well.

Our PSII model also proposes the presence of up to three monomeric Lhc subunits (Figures 2 and 4). Assuming that these subunits are specific for PSII and thus not part of the trimeric pool (Grouneva et al., 2011; Nagao et al., 2013; Gundermann et al., 2019), two FCP polypeptides have been identified in PSII (Calvaruso et al., 2020) that can be safely attributed: Lhca2 and Lhcf11. The amino acid sequence of *T. pseudonana* Lhca2 agrees with the sequence of FCP-D monomer in *C. gracilis* (Pi et al., 2019). Due to the absence of the Lhca2 structure, we used the structure of FCP-D (Pi et al., 2019) to fit the density of FCPII-1 monomer in the PSII model (Figure 2). We propose assigning Lhcf11 to the FCPII-2 position, although the localization of this protein at the FCPII-3 position cannot be ruled out. The question remains about the identity of FCPII-3. Based on a smaller size of the FCPII-3 density, we fitted FCPII-3 with PtLhcf4 from *Phaeodactlyum tricornutum* (Wang et al., 2019), the most compact structure of an FCP reported so far. In terms of amino acid sequence, *Pt*Lhcf4 does not match unequivocally with any *T. pseudonana* protein, but it is still rather similar to most *Tp*Lhcf. Therefore, we propose the FCPII-3 most probably also belongs to the Lhcf group.

### Structure of the PSI supercomplex in *T. pseudonana*

The structural model of the PSI supercomplex of *T. pseudonana* shows a PSI core surrounded by a large number of FCPI subunits, which is in agreement with the structure of PSI observed in *C. gracilis* (Nagao et al., 2020a, 2020b; Xu et al., 2020). However, our two models do not completely agree on the number of individual FCPI subunits present in the light-harvesting antenna. Whereas the model obtained by cryo-ET predicts that the PSI core is surrounded by 18 FCPI subunits (Figure 3), in the structural analysis of isolated PSI particles we have identified 20 FCPI subunits (Figure 4, C and D). This discrepancy could be probably explained by the fact that EM tomography has a lower resolution than the EM single-particle analysis and thus it could have underestimated the number of FCPI. Nevertheless, the two structural models that are already available for PSI-FCPI in *C. gracilis* (Nagao et al., 2020a, 2020b; Xu et al., 2020) also do not agree on the detailed composition of the PSI antenna, the predicted number of individual FCPI subunits ranging between 16 and 24. A different number of FCP subunits associated to PSI in *T. pseudonana* and *C. gracilis* can indicate either a flexible adjustment of the FCPI moiety under different growth conditions (Nagao et al., 2020a, 2020b) or a loose binding of individual FCPI subunits to PSI complex, which can lead to their loss during isolation. The latter factor is likely responsible for the structural variability of PSI supercomplexes often observed in other species of lower plants as algae and mosses (see, e.g. Pinnola et al., 2018; van den Berg et al., 2020), which, in general, possess a larger antenna size compared to vascular plants. A smaller antenna size of PSI from vascular plants, which typically consists of only four Lhca1-4 proteins (Ben-Shem et al., 2003), is structurally more stable during isolation procedure.

The presence of membrane-extrinsic densities at both stromal and luminal sides of the cryo-ET map of PSI indicates that we were able to capture the binding of soluble electron carriers to the core complex. Recently, a cryo-EM structure of a triple complex of plant PSI with Pc and ferredoxin revealed information about the mechanism of the binding of electron carriers to PSI (Caspy et al., 2020). The binding of Pc was shown to depend on the interactions with PsaA, PsaB, and PsaF subunits. This arrangement is in line with our 3D map of PSI that exhibits the densities at the donor site, thus indicates a very similar binding of cyt *c6* to PSI in *T. pseudonana* (Figure 3D).

Considering the size of the extrinsic density on the acceptor side of PSI in our cryo-ET map, we assume that our model managed to capture the binding of both ferredoxin and ferredoxin NADP^+^ reductase (FNR) to PSI. The recent plant cryo-EM structure (Caspy et al., 2020) revealed that ferredoxin at the acceptor side seems to be attached to PSI via electrostatic interactions mediated by PsaA, PsaC, and PsaE subunits. This is in agreement with several previous biochemical studies focused on the identification of PSI subunits involved in the binding of FNR. In barley plants, cross-linking and western blotting experiments revealed that FNR associates with PSI core complex through PsaE (Andersen et al., 1992). In *C. reinhardtii*, isothermal titration calorimetric experiments showed that, in addition to PsaE, the binding site of FNR is shared between PsaB, PsaF, and inner antenna subunits of the LHCI belt (Marco et al., 2019). Our 3D map of PSI indicates that the binding sites of FNR seem to be the same in *T. pseudonana*. In conclusion, the sub-tomogram averaged map of PSI in *T. pseudonana* represents a huge “electron transfer protein complex” consisting of PSI with cyt *c_6_*, ferredoxin and FNR (Figure 3, C and D), whose binding shares similar features with other photosynthetic organisms.

### Spatial segregation of photosynthetic protein complexes in the thylakoid membrane

The resolution of the cryo-ET techniques enabled us to distinguish densities representing various protein complexes in the thylakoid membrane, but per se it did not allow their unequivocal identification. However, thanks to the subsequent sub-volume averaging, we were able to identify individual densities either as PSII or PSI supercomplexes or as ATP synthase. Tracking back the location of individual complexes in the thylakoid membrane then revealed their spatial segregation (Figure 5). It appears that the majority of PSII supercomplexes are confined to the central region of the membrane vesicle, whereas both PSI and ATP synthase are located preferentially at the edge. This is in agreement with a generally accepted model of spatial segregation of PSs in the thylakoid membrane of photosynthetic organisms (Dekker and Boekema, 2005). Our data confirm, in line with other studies on diatoms (Flori et al., 2017; Levitan et al., 2019), that the spatial segregation of components of the photosynthetic apparatus is a key concept in the optimization of photosynthetic function.

Interestingly, a similar distribution of PSII, PSI, and ATP synthase particles was observed on both sites of the vesicle (Figure 5). Stroma exposed subunits of PSI and the hydrophilic head of ATP synthase would preclude a stacking of the thylakoid vesicle with the adjacent thylakoids. However, tracking of the thylakoid membrane in the reconstructed tomogram revealed a membrane opening in the central part of the vesicle (Supplemental Movie S1). This membrane protrusion most probably represents a residual part of one of the connections to the neighboring thylakoid, which were previously observed between parallel layers of stacked thylakoids in diatoms (Flori et al., 2017). This thylakoid structure resembles the so-called anastomoses observed in the thylakoid membrane of *P. tricornutum* (Flori et al., 2017). As the height of this protrusion is approximately 40 nm, it can prevent close contact between the two neighboring thylakoid vesicles, which would enable the distribution of PSI and ATP synthase particles in both layers of the thylakoid vesicle. However, these data have to be interpreted with caution, as the original architecture of these anastomoses might have been changed during the destacking treatment that was necessary for the preparation of the cryo-ET samples.

### Structural diversity of diatom photosynthetic complexes

In this work, PS structures of the diatom *T. pseudonana* are described for the first time. The presented models confirm the heterogeneity in the diatoms photosynthetic apparatus. Recent works revealed the diversity of Lhc structure in different diatom species (Wang et al., 2019; Röding et al., 2018; Nagao et al., 2019; Pi et al., 2019; Nagao et al., 2020a, 2020b; Xu et al., 2020): dimeric FCP was found in *P. tricornutum*, tetrameric in *C. gracilis* and trimeric complexes in the centric diatom *Cyclotella meneghiniana*. This diversity was also observed at biochemical level, employing native gel electrophoresis methods (Nagao et al., 2020a, 2020b).

Although all the species belong to the same algal group, some differences can be found (Supplemental Figure S3, B and C). One major distinction concerns the pigment composition of their light-harvesting antennae. High-performance liquid chromatography (HPLC) analysis of the PSII-SG fraction from *T. pseudonana* found different Chl stoichiometries compared to *C. gracilis* PSII. A smaller Chl c:a ratio was observed for *T. pseudonana* PSII supercomplexes, which is in line with the fact that *C. gracilis* FCPs are enriched in Chl c (3:7 Chl c:a) compared to other diatom species such as *Phaoedactylum tricornutum* and the species most closely related to *T. pseudonana*, *C. meneghiniana* (2:7 Chl c:a; Gundermann et al., 2019; Wang et al., 2019). As FCPs arrangement should optimize the energy transfer to the reaction center, different pigment ratios may result in a different protein organization—indeed, FCP trimers were found in *T. pseudonana* as opposed to the tetramers present in *C. gracilis*. Another difference between these two species is the enrichment of certain xanthophylls in the PSII SG fraction from *T. pseudonana* (Supplemental Figure S3C). Xanthophylls are a group of carotenoids also involved in photoprotection; in diatoms mainly diadinoxanthin and diatoxanthin constitute the xanthophyll cycle needed for this function. Diadinoxanthin is enriched in the PSII fraction analyzed here, whereas it is poorly present in *C. gracilis* PSII. This enrichment in xanthophyll cycle pigments might be related to the presence of Lhcx6_1 within the *T. pseudonana* PSII supercomplex (Supplemental Figure S3B). A similar subunit was not demonstrated for *C. gracilis* PSII, where all Lhc were fitted with either Lhcf or Lhcr proteins. Xanthophyll cycle pigments and Lhcx proteins are involved in the nonphotochemical quenching mechanisms in diatoms, but unfortunately, the precise molecular aspects are still unknown for both *C. gracilis* and *T. pseudonana*. Therefore, the PSII variability might also be based on a different photoprotective strategy adopted by the two species, possibly involving Lhcx proteins directly attached to PSII only in the case of *T. pseudonana*.

Comparing available PS structures from *T. pseudonana* and *C. gracilis*, one fact looks clear: structural heterogeneity affects mostly PSII, whereas PSI is more conserved. It is possible that the higher diversity of diatom PSII compared to PSI is closely linked to the mechanism of their origin. Diatoms originated from a secondary endosymbiotic event between a red algal ancestor and an eukaryotic cell (Bhattacharya et al., 2007), that is, the common ancestor of all diatoms had to rely on the photosynthetic machinery of red algae. Comparison of the diatom PSI complex (Nagao et al., 2020a, 2020b; Xu et al., 2020) with a recently obtained structure of PSI in a red alga (Pi et al., 2018) suggests that the PSI complex did not undergo any extensive changes since that time. Therefore, it is reasonable to assume that the overall PSI architecture taken over from the red alga was optimal also for the newly formed organisms and thus it was conserved without substantial changes up today. With PSII, the situation seems to be entirely different. Red algae are known to rely on phycobilisomes as the PSII-specific antennae (Gantt et al., 2003), which are, however, completely absent in diatoms. The introduction of a new, much more modular PSII antenna seems to have triggered a huge variety of systems, reflected on one hand in the much higher number of different FCP subunits as compared to land plants, but on the other hand also by different structural arrangements inside the PSII supercomplexes. This variety probably enables extreme adaptability, which might be one of the reasons for the huge ecological success of this important group of phytoplankton species.

## Materials and methods

### *Thalassiosira pseudonana* cultivation and isolation of intact plastids

*Thalassiosira pseudonana* cells (strain CCMP1335, Hustedt) were cultivated in f/2 medium (Guillard, 1975), with a light intensity of 45 µmol photons s^−1^ m^−2^. Cultures were maintained at 15°C and under a 16–8 h day–night cycle. As a starting material for the plastid isolation (Schober et al., 2018), 4 L of cell culture was prepared and cells were harvested after 5 d, at a final concentration of 4–6 × 10^6^ cells mL^−1^. Equipment and solutions were kept at 4°C and the preparation was performed in semi-darkness. After harvest (5,000*g*, 10 min), the cells were washed with isolation medium (0.5 M sorbitol, 50 mM HEPES-KOH, 6 mM Na-EDTA, 5 mM MgCl_2_, 10 mM KCl, 1 mM MnCl_2_, 1% (w/v) polyvinylpyrrolidone 40, pH 7.4), supplied with 0.5% (w/v) defatted BSA and 0.1% (w/v) L-cysteine. Before use, the osmolality of the isolation medium was adjusted to 750 ± 20 mOsm kg^−1^. After washing, cell material was centrifuged (2,500*g*, 10 min), resuspended in 12 mL isolation medium with supplements, and the cells were opened by French Press treatment (two cycles at 14.5 MPa). After the disruption, a centrifugation step (300*g*, 9 min) was applied to separate unbroken cells (pellet) from free plastids (supernatant), which were then pelleted by applying 6,000*g* for 10 min and gently resuspended in 2–4 mL of isolation medium without supplements. Once dissolved, the sample was applied onto a discontinuous Percoll gradient (10–20–30% (v/v) phases, in isolation medium) and run 30 min at 14,400*g* with an ultracentrifuge Sorvall Discovery SE90 (swing-out rotor AH-629; Thermo Fisher Scientific, Waltham, MA). Plastids were collected between the 20% and 30% gradient steps. To remove the Percoll, plastids were washed using an isolation medium and then concentrated by another centrifugation step (4,000*g*, 10 min)*.* Afterwards, Chl *a* concentration was determined in 90% (v/v) acetone (Jeffrey and Humphrey, 1975). Chlorophyll concentration was adjusted to 1.5 µg/µL of Chl *a*, and aliquots were flash-frozen in liquid nitrogen and stored at −80°C.

### Preparation of unstacked thylakoid membranes

*Thalassiosira pseudonana* intact plastids were partially unstacked to obtain single thylakoid units. The destacking was performed using a buffer without Mg^2+^ and a low concentration of detergent (Jäger and Büchel, 2019): a mixture containing 50 µL of membrane suspension, 1 mL of buffer (50 mM HEPES-KOH, 6 mM EDTA), and 0.01% (w/v) n-dodecyl-α-D-maltoside (α-DDM) was incubated overnight in the dark and at 4°C. After incubation and centrifugation (5,000*g*, 10 min), thylakoid membranes were collected from the pellet and resuspended in 70 µL of the abovementioned buffer.

### Purification of PSII and PSI supercomplexes

Native PSII and PSI supercomplexes were isolated by SG ultracentrifugation according to Calvaruso et al. (2020). Plastids were washed in MMKB buffer (30 mM 2-[N-morpholino] ethanesulfonic acid, 5 mM MgCl_2_, 10 mM KCl, 1 M betaine, pH 6.5) and incubated for 30 min at 4°C in MMKB buffer with 0.75% (w/v) α-DDM. After removing insolubilized material (17,383*g*, 1 min), complexes were separated by SG ultracentrifugation (132,000*g*, 16 h, 4°C). SG was prepared by freeze–thaw cycles of MMKB buffer supplied with 0.55 M sucrose and 0.03% (w/v) α-DDM. After the run, SG bands were harvested and stored at −80°C, after flash-freezing in liquid nitrogen. For biochemical analysis, SG fractions were concentrated using Amicon-ultra devices (Merck-Millipore, Burlington, MA, USA) with a molecular weight cut-off of 100 kDa and then stored at −80°C before use.

### Biochemical analysis of SG fractions

Blue-native (BN) polyacrylamide gel electrophoresis (PAGE; Järvi et al., 2011) was employed to evaluate the composition of protein complexes in the SG fractions. 2.5 µg Chl *a* per fraction were loaded onto acrylamide gradient gel, 3–8% (v/v), after adding 3.7 µL loading buffer (100 mM BisTris/HCl, pH 7.0, 0.5 M ε-aminocaproic acid, 30% (w/v) sucrose and 50 mg mL^−1^ Serva Blue G). Samples were run at 4°C and 6 mA (max. 150 V) with BN cathode buffer (50 mM Tricine, 15 mM BisTris/HCl, pH 7.0) with 0.01% (w/v) Serva Blue G for 1.5 h. The run was then completed overnight in cathode buffer without Coomassie using 50 V fixed voltage. For both steps, BN anode buffer was used (50 mM BisTris/HCl, pH 7.0). To analyze the subunit composition (Calvaruso et al., 2020), PSI/PSII-containing bands were excised from the BN gel and their peptides were resolved via 2D-SDS-PAGE using a Tris–Tricine gel system. After the run, the 2D-gel was silver stained (Blum et al., 1987) or used for western blot analysis. Proteins were electroblotted onto a polyvinylidenfluorid membrane, which was then incubated for 1 h (or overnight) with 5% (w/v) skim milk in phosphate-buffered saline buffer (137 mM NaCl, 2.7 mM KCl, 10 mM Na_2_HPO_4_, 1.8 KH_2_PO_4_, pH 7.4) and 0.1% (w/v) Tween 20. After the primary and secondary antibody incubation (1 h at room temperature, with three washing steps after each incubation), the membrane was developed by enhanced chemiluminescence method (Alegria-Schaffer et al., 2009). Primary antibodies were: α-cmFCP (Juhas and Büchel, 2012; directed mainly against the pool of Lhcf proteins, diluted 1:5,000), α-FCP2, and α-FCP4 (Westermann and Rhiel, 2005; directed against TpLhcf1/2 and TpLhcr4 respectively, diluted 1:2,000) and α-PtLhcf1-11 (Juhas and Büchel, 2012; highest affinity to TpLhcf8/9, diluted 1:2,000). The antibody α-Lhcx6_1 (diluted 1:5,000), recognizing the Lhcx6_1 polypeptide sequence, was obtained by commercial production of antiserum (Eurogentec, Seraing, Belgium) using a synthesized peptide NH_2_-DKPLLVNLQDSGFVSWC-COOH. The same secondary antibody was used for all the experiments: goat anti-rabbit specific peroxidase conjugate (Calbiochem catalog no. 401315, diluted 1:10,000).

HPLC was performed for the pigment analysis of the SG bands. Pigment extract in 90% acetone (v/v) was separated by a reversed-phase column (Lichrosorb RP18, 5 mm, 250 × 4 mm) and HPLC chromatograms were used for the analysis of the elution profiles and pigment quantification (Papagiannakis et al., 2005).

### Cryo-electron tomography

For the preparation of cryo specimen, 4 µL of unstacked thylakoid membrane suspension were mixed with an equal volume of 10 nm gold fiducial markers (BSA tracer; AURION, Wageningen, The Netherlands) and applied on a Quantifoil grid (3.1/1, Cu, 200 mesh; Quantifoil) coated with 15 nm of amorphous carbon film. Grids were incubated at 25°C for 1 min in a 100% humidified chamber of Vitrobot (Mark IV, FEI) before they were plunge frozen in liquid ethane, cooled by liquid nitrogen. Single-axis tilt series of the cryo samples were imaged in a Titan Krios microscope (Thermo Fisher Scientific) equipped with Gatan energy filter and K2 direct electron detector (Gatan). Tilt series ranging from −64° to +64° at an increment step of 2° were recorded using SerialEM software (Mastronarde, 2005) at ×53,000 magnification with specimen level pixel size of 2.84 Å. The electron dose was set between 50 and 60 e/Å^2^.

### Tomography data processing

Tilt series were processed in a semi-automated manner for the generation of the tomograms using IMOD software package (Kremer et al., 1996). The alignment of raw images was performed using gold fiducials as markers for cross-correlation. Tomograms were binned 4× for enhancing the contrast and further denoised by iterative nonlinear anisotropic diffusion.

Sub-volume averaging was performed in EMAN2 (Tang et al., 2007; Electron Micrograph Analysis 2) software. Sub-volumes containing densities of PSII, PSI, and ATP synthase were manually picked from the tomographic slices. In total, 578, 374, and 322 sub-volumes of PSII, PSI, and ATP synthase, respectively, were analyzed. After removing bad particles by using a cross-correlation threshold, the data were subjected to the 3D refinement and final maps were obtained. The resolution of obtained 3D maps was determined by gold standard Fourier shell correlation, which was found to be 4.2, 3.3, and 3.4 nm (cutoff 0.143) for PSII, PSI, and ATP synthase, respectively. The 3D map of PSII core and associated minor antenna proteins were fitted with the *C. gracilis* high-resolution structure (PDB 6 jlu), whereas trimeric antenna proteins were fitted with pea (*Pisum sativum*) LHCII trimer (PDB 2 bhw). The sub-averaged 3D map of PSI was fitted with the *C. gracilis* structure (PDB 6 ly5), and the ATP synthase map was fitted with spinach chloroplast ATP synthase structure (PDB 6 fkh). Additional densities at the stromal side of the PSI complex were fitted by the crystal structure of the maize complex between FNR (PDB 1 gaq) and the luminal densities of PSI were fitted using cyt c_6_ (PDB 3dmi). The models were built by a fitting of multiple subunit rigid bodies of pdb models into the density maps. A membrane segmentation model was created by mapping back the coordinates of the PSII, PSI, and ATP synthase sub-volumes to the original tomogram. Sub-volumes from three refinements (corresponding to PSII, PSI, and ATP synthase) were back-projected in the tomogram and visualized using UCSF Chimera (Pettersen et al., 2004; University of California, San Francisco). Isosurface thresholds were adjusted to the best visualization of reconstructed volumes and tomogram was translated and rotated along *x*, *y*, and *z* planes for generating a segmentation model and a movie.

### EM of isolated PSII and PSI supercomplexes

Samples from SG fractions were dialyzed at 4°C for 4 h using a 14 kDa cut-off dialysis membrane (Carl-ROTH, Karlsruhe, Germany). Sucrose-free, isolated supercomplexes were subjected to negative staining with 2% uranyl acetate on glow-discharged carbon-coated copper grids. Electron microscopic data were collected using Tecnai G2 F20 microscope (FEI) with an Eagle 4K CCD camera (FEI). Images of 2,048 × 2,048 pixels were recorded at ×133,000 magnification with a pixel size of 0.226 nm. EPU software (FEI) was used for the automated acquisition of 6,141, 10,410, and 11,043 micrographs from PSII, PSI, and FCPs fractions, respectively. From the collected images 121,067, 98,280, and 405,837 single-particle projections of PSII, PSI, and FCPs, respectively, were independently picked and analyzed. After initial sorting, particles were subjected to reference-free 2D alignment and classification using SCIPION image processing framework (De La Rosa-Trevín et al., 2016). Resolution of the PSII and PSI projection maps was determined by Fourier ring correlation using Relion software (Scheres, 2012) and it was found to be 19 and 17 Å, respectively.

## Supplemental data

The following materials are available in the online version of this article.

**Supplemental Figure S1.** Particle picking and averaged 3D volumes of PSII, PSI, and ATP synthase.

**Supplemental Figure S2.** 2D projection and fitting of trimeric light-harvesting antenna.

**Supplemental Figure S3.** Biochemical analysis of the PS SG fractions.

**Supplemental Figure S4.** Structural models of PSII supercomplexes from *T. pseudonana* revealed by EM and single-particle analysis.

**Supplemental Figure S5.** Structural models of PSI supercomplexes from *T. pseudonana* revealed by EM and single-particle analysis.

**Supplemental Figure S6.** Comparison of core-antenna organization of PSII supercomplexes.

**Supplemental Movie S1.** Slice by slice view of the tomogram and tracking of the membrane.

**Supplemental Movie S2.** Sub-tomogram averaged volume of PSII.

**Supplemental Movie S3.** Sub-tomogram averaged volume of PSI.

**Supplemental Movie S4.** Special distribution of the components of thylakoid membrane.

## Supplementary Material

kiab208_Supplementary_Data

## Notes

R.A., C.C., C.B., and R.K. conceived the project. C.C. purified plastids, the PSII and PSI supercomplexes, FCP complexes, and performed their biochemical and pigment analysis. R.A. collected cryo-electron tomography tilt series and electron micrographs of isolated PSII, PSI supercomplexes, and FCP complexes. R.A. and R.K. processed the EM data and built the structural models of PSII and PSI supercomplexes. R.A. built a model of reconstructed tomogram and final movies. R.A., C.C., C.B., E.J.B., and R.K. proposed interpretation of the structural data. R.A., C.C., C.B., and R.K. wrote the paper. All of the authors contributed to the interpretations of the results and improvement of the manuscript.

The author responsible for distribution of materials integral to the findings presented in this article in accordance with the policy described in the Instructions for Authors (https://academic.oup.com/plphys/pages/general-instructions) is: Roman Kouřil (roman.kouril@upol.cz).
