# Supplementary material for: Revealing the architecture of the photosynthetic apparatus in the diatom Thalassiosira pseudonana
Source: Plant Physiol. 2021 May 4;186(4):2124–36. doi: 10.1093/plphys/kiab208 (PMC8331139; doi:10.1093/plphys/kiab208)
Supplement: kiab208_Supplementary_Data [file kiab208_supplementary_data.zip › Supplemental data-edited-6-May-2021.pdf]

Supplemental Data

Supplemental Figures

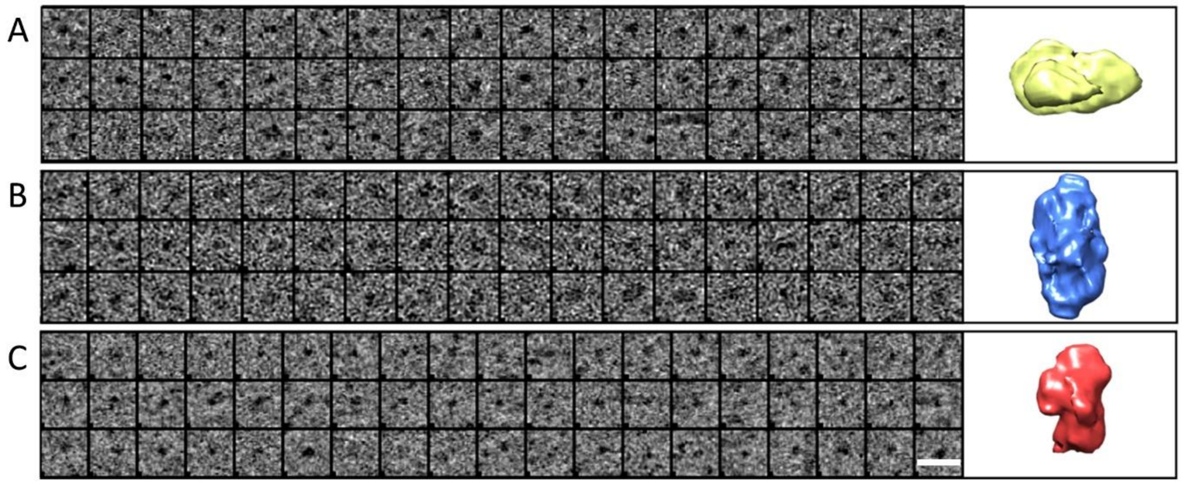

Supplemental Figure S1. Particle picking and averaged 3D volumes of PSII, PSI and ATP synthase. A-C, 2D boxing of the particles selected from the slices of membrane tomogram. Particles were extracted and subjected to 3D alignment to obtain sub-averaged volumes. 3D volumes of PSII, PSI and ATP synthase are shown in blue, yellow and red, respectively. Contrast of the picked particles is enhanced for visualization. The scale bar is 30 nm.

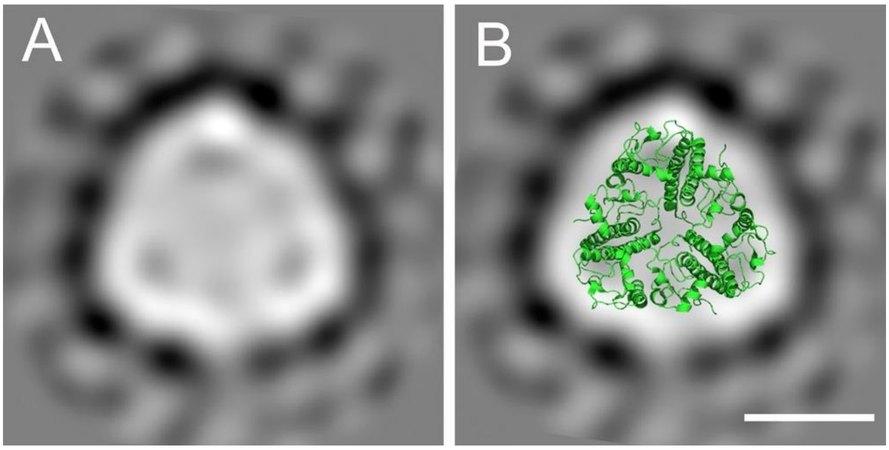

Supplemental Figure S2. 2D projection and fitting of trimeric light-harvesting antenna. A, a 2D class obtained from single particle analysis of negatively stained SG samples representing a sum of 3,449 projections of trimeric form of light-harvesting antenna. B, Overlap of pea LHCII trimer (PDB 2bhw) with the EM densities. The scale bar is 10nm.

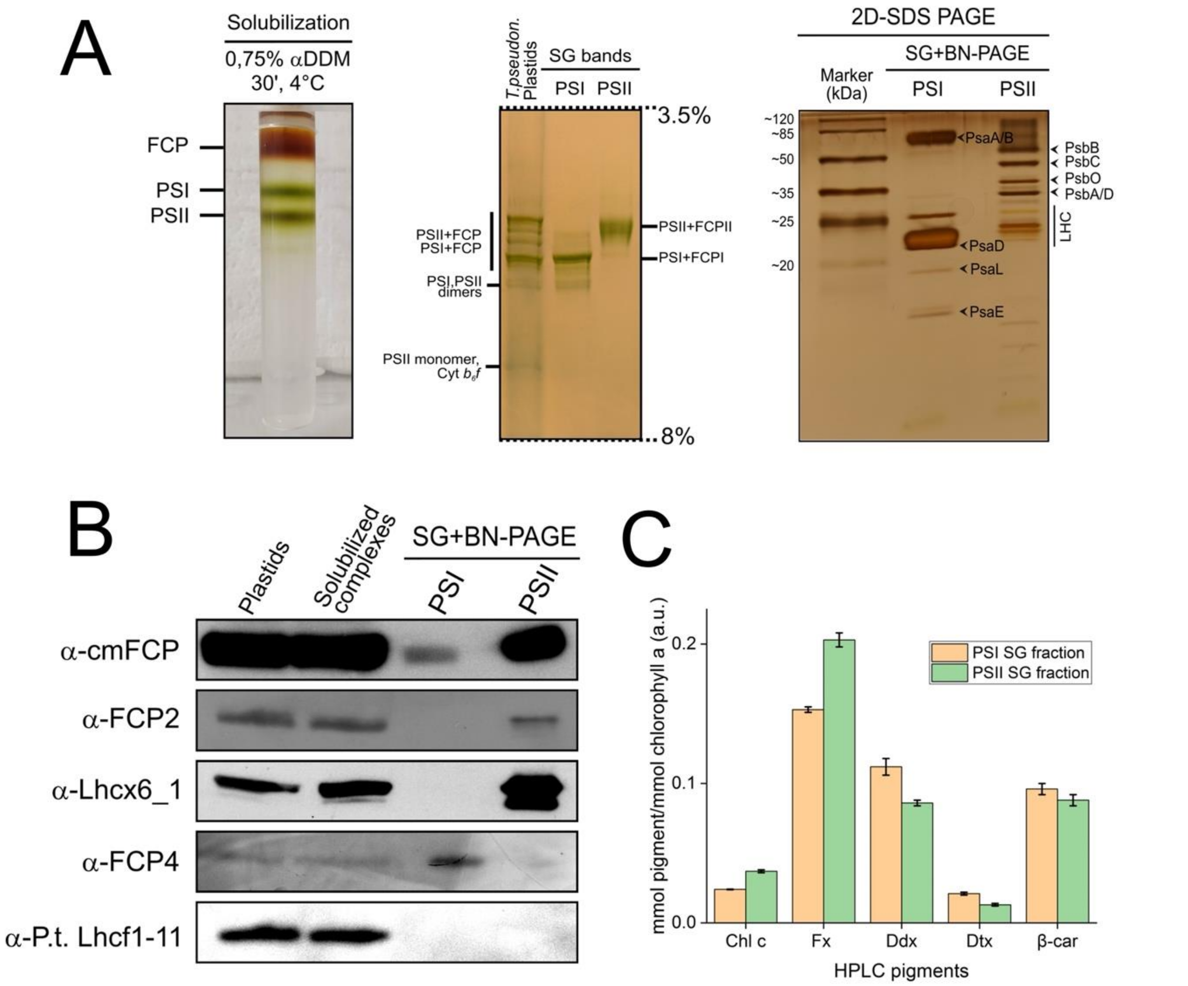

Supplemental Figure S3. Biochemical analysis of the photosystem SG fractions. A, Complexes were separated by SG centrifugation (left). Using BN-PAGE (middle), the two SG fractions containing photosystems were further analyzed and the most intense bands were used for 2D-SDS-PAGE (right). B, analysis of the antenna composition of the photosystems by western blot. From left to right: Intact plastids, solubilized native complexes (incubation 30 min in 0.75% (w/v) α-DDM), photosystems obtained by SG followed by BN-PAGE. The double purification step was used to avoid any cross-contamination occurring during the harvest of the SG fractions. For detection of the pool of Lhcf proteins α-cmFCP (Juhas and Büchel, 2012) was used, TpLhcf1/2 was detected by α-FCP2, TpLhcr using α-FCP4 (Westermann and Rhiel, 2005), and TpLhcf8/9 was visualized using α-PtLhcf1-11 (Juhas and Büchel, 2012). C, Pigment composition of the photosystems isolated by SG. Values are averages between two independent biological replicates (n=2), with three technical replicates each. Error bars represent average of standard deviations calculated from technical replicates.

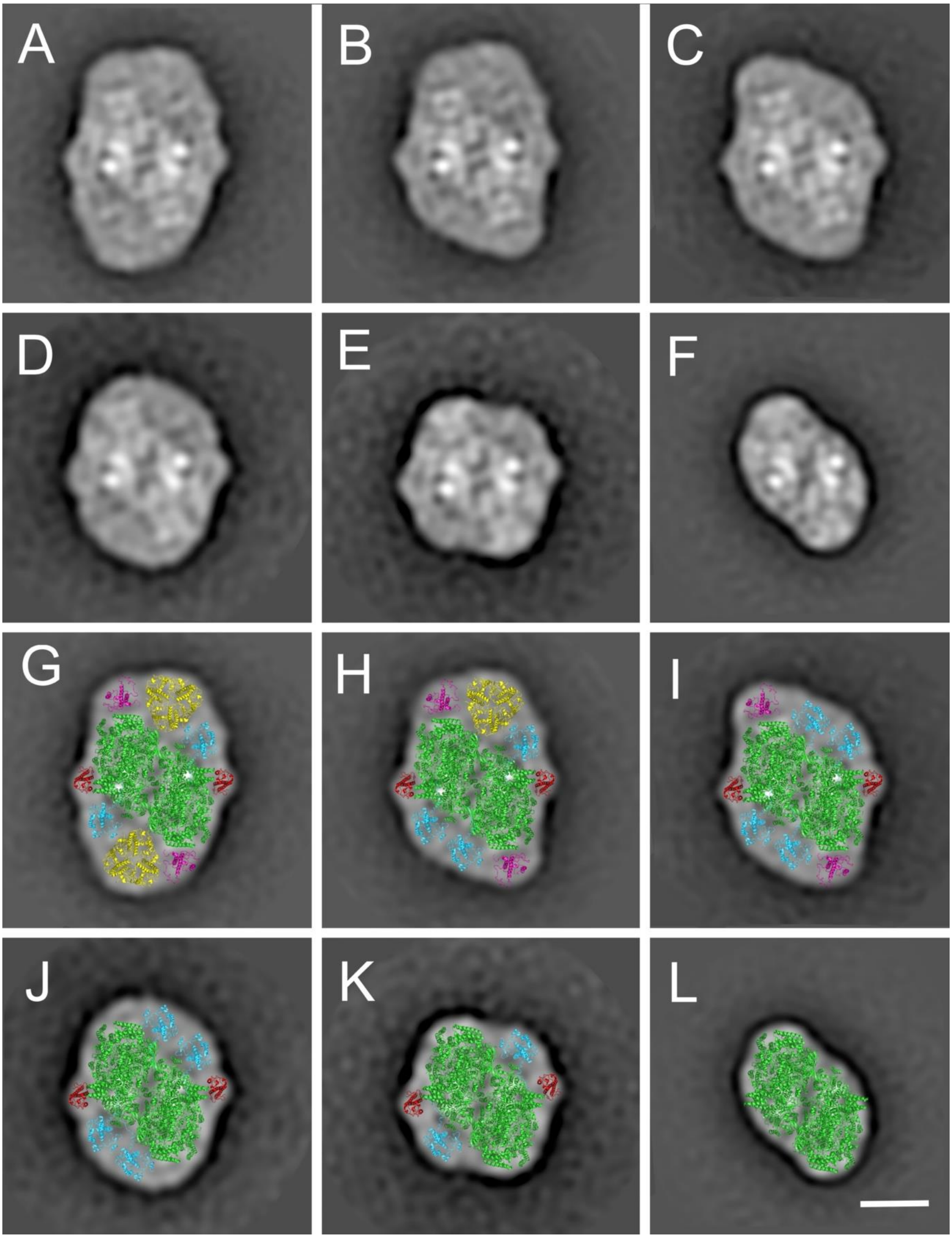

Supplemental Figure S4. Structural models of PSII supercomplexes from *Thalassiosira pseudonana* revealed by electron microscopy and single particle analysis. A-F, averaged 2D projections of negatively stained PSII supercomplexes with a class sum of 3,417, 4,843, 4,134, 3,404, 1,440 and 7,833 particles, respectively. G-L, assignment of EM densities based

on the fitting with high-resolution structures. Densities representing PSII core complex and monomeric antenna are fitted with the PSII structure from *C. gracilis* (PDB 6jlu). The trimeric form of light-harvesting antenna is fitted with pea LHCII structure (PDB 2bhw). PSII densities close to PsbX were fitted with *P. tricornutum* FCP monomer (PDB 6a2w). PSII core complex is shown in green, FCPII-1 in cyan, FCPII-2 in purple, FCPII-3 in red and trimeric FCP antenna (FCPII-S) is in yellow color. The scale bar is 10 nm.

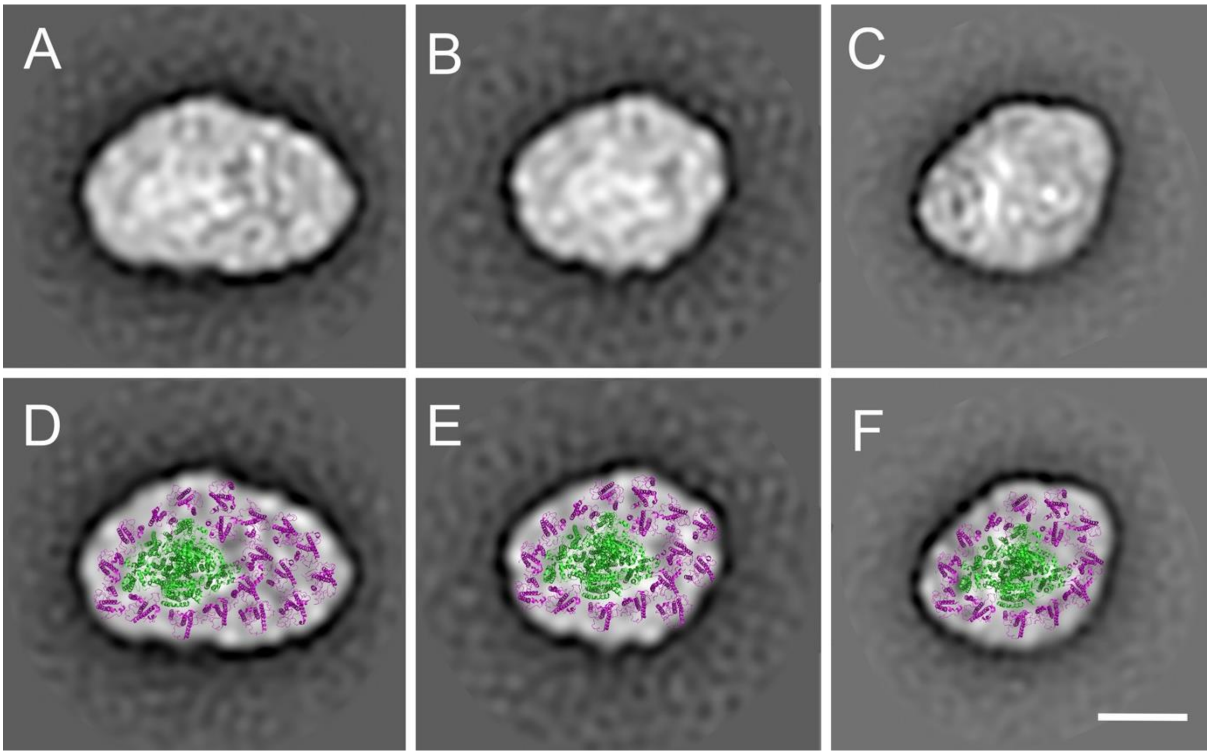

Supplemental Figure S5. Structural models of PSI supercomplexes from *Thalassiosira pseudonana* revealed by electron microscopy and single particle analysis. A-C**,** averaged 2D projections with class sums of 1,155, 2,022 and 1,116 particles, respectively. D-F, assignment of EM densities is based on the fitting with high-resolution structure from *C. gracilis* (Xu et al., 2020) (PDB 6ly5). The PSI core complex is indicated in green and FCP subunits are in purple color. The scale bar is 10 nm.

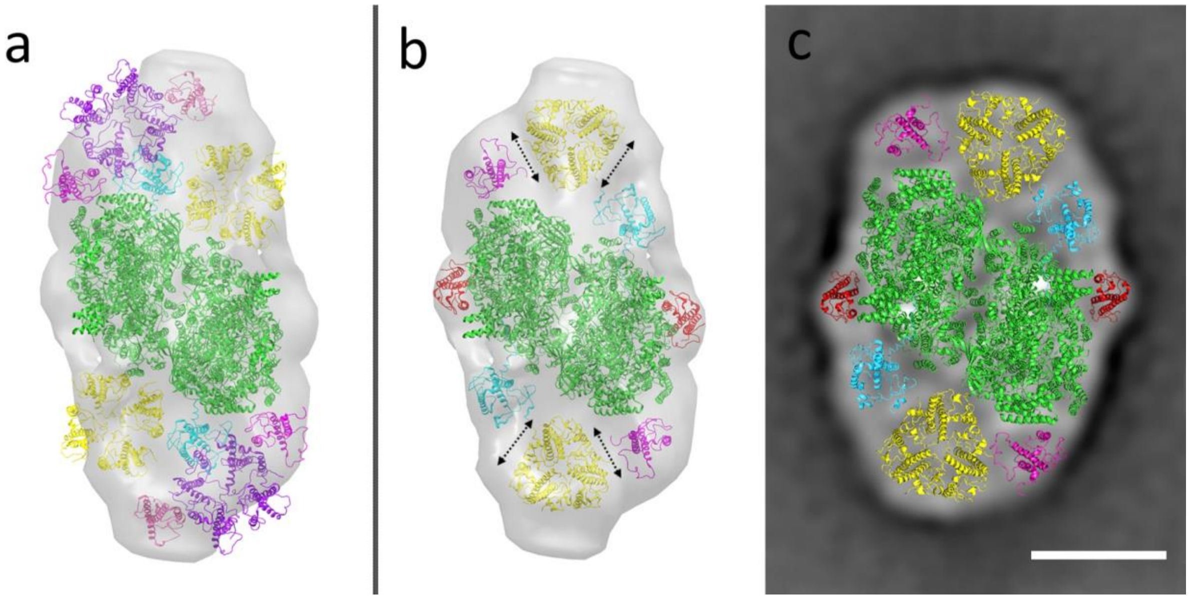

Supplemental Figure S6. Comparison of core-antenna organization of PSII supercomplexes. A, overlay of PSII model from *C. gracilis* on the sub-volume of *T. pseudonana*. B, 3D map of PSII obtained by sub-averaging of PSII projections picked from membrane tomogram. Black arrows indicate the lateral shift of trimeric light-harvesting antenna in tomographic model compared to the arrangement visible in single particle analysis. C, 2D projection map of PSII generated by single particle analysis of isolated supercomplexes. The scale bar is 10 nm. Densities representing PSII core complex and monomeric antenna are fitted with the PSII structure from *C. gracilis* (PDB 6jlu). The trimeric form of light-harvesting antenna is fitted with pea LHCII structure (PDB 2bhw). Novel PSII densities close to PsbX were fitted with *P. tricornutum* FCP monomer (PDB 6a2w). PSII core complex is shown in green, FCPII-1 in cyan, FCPII-2 in purple, FCPII-3 in red and trimeric FCP antenna (FCPII-S) is in yellow color.

Supplemental movies

Supplemental Movie S1. Slice by slice view of the tomogram and tracking of the membrane.

Supplemental Movie S2. Sub-tomogram averaged volume of PSII.

Supplemental Movie S3. Sub-tomogram averaged volume of PSI.

Supplemental Movie S4. Special distribution of the components of thylakoid membrane.
